# Environmental Applications of Biosurfactants: Recent Advances

**DOI:** 10.3390/ijms12010633

**Published:** 2011-01-18

**Authors:** Magdalena Pacwa-Płociniczak, Grażyna A. Płaza, Zofia Piotrowska-Seget, Swaranjit Singh Cameotra

**Affiliations:** 1 Department of Microbiology, Silesian University, Jagiellońska 28 street, 40-032 Katowice, Poland; E-Mails: mpacwa@us.edu.pl (M.P.-P.); zofia.piotrowska-seget@us.edu.pl (Z.P.-S.); 2 Department of Environmental Microbiology, Institute for Ecology of Industrial Areas, Kossutha 6 street, 40-844 Katowice, Poland; 3 Institute of Microbial Technology, Sector 39A, Chandigarh-160036, India; E-Mail: ssc@imtech.res.in

**Keywords:** biosurfactants, hydrocarbons, metals, remediation technologies

## Abstract

Increasing public awareness of environmental pollution influences the search and development of technologies that help in clean up of organic and inorganic contaminants such as hydrocarbons and metals. An alternative and eco-friendly method of remediation technology of environments contaminated with these pollutants is the use of biosurfactants and biosurfactant-producing microorganisms. The diversity of biosurfactants makes them an attractive group of compounds for potential use in a wide variety of industrial and biotechnological applications. The purpose of this review is to provide a comprehensive overview of advances in the applications of biosurfactants and biosurfactant-producing microorganisms in hydrocarbon and metal remediation technologies.

## 1. Introduction

Biosurfactants are a structurally diverse group of surface-active substances produced by microorganisms. All biosurfactants are amphiphiles, they consist of two parts—a polar (hydrophilic) moiety and non polar (hydrophobic) group. A hydrophilic group consists of mono-, oligo- or polysaccharides, peptides or proteins and a hydrophobic moiety usually contains saturated, unsaturated and hydroxylated fatty acids or fatty alcohols [1]. A characteristic feature of biosurfactants is a hydrophilic-lipophilic balance (HLB) which specifies the portion of hydrophilic and hydrophobic constituents in surface-active substances.

Due to their amphiphilic structure, biosurfactants increase the surface area of hydrophobic water-insoluble substances, increase the water bioavailability of such substances and change the properties of the bacterial cell surface. Surface activity makes surfactants excellent emulsifiers, foaming and dispersing agents [2]. In comparison to their chemically synthesized equivalents they have many advantages. They are environmentally friendly, biodegradable, less toxic and non-hazardous. They have better foaming properties and higher selectivity. They are active at extreme temperatures, pH and salinity as well, and can be produced from industrial wastes and from by-products. This last feature makes cheap production of biosurfactants possible and allows utilizing waste substrates and reducing their polluting effect at the same time [3–7].

Because of their potential advantages, biosurfactants are widely used in many industries such as agriculture, food production, chemistry, cosmetics and pharmaceutics. The examples of biosurfactant applications are listed in many review papers [8–10]. In this review, special attention is paid to the use of biosurfactants in different aspects of environmental biotechnology. Many properties of microbial surface active compounds such as emulsification/de-emulsification, dispersion, foaming, wetting and coating make them useful in physico-chemical and biological remediation technologies of both organic and metal contaminants. Biosurfactants increase the bioavailability of hydrocarbon resulting in enhanced growth and degradation of contaminants by hydrocarbon-degrading bacteria present in polluted soil. In heavy-metal polluted soils biosurfactants form complexes with metals at the soil interface, which is followed by desorption of the metal and removal from the soil surface leading to the increase of metal ions concentration and their bioavailability in the soil solution. The new approach is the use of heavy metal-resistant bacterial strains capable of producing biosurfactants for increasing the metal-removing efficiency by phytoremediation.

## 2. Classification and Properties of Biosurfactants

Unlike chemically synthesized surfactants, which are classified according to their dissociation pattern in water, biosurfactants are categorized by their chemical composition, molecular weight, physico-chemical properties and mode of action and microbial origin. Based on molecular weight they are divided into low-molecular-mass biosurfactants including glycolipids, phospholipids and lipopeptides and into high-molecular-mass biosurfactants/bioemulsifiers containing amphipathic polysaccharides, proteins, lipopolysaccharides, lipoproteins or complex mixtures of these biopolymers. Low-molecular-mass biosurfactants are efficient in lowering surface and interfacial tensions, whereas high-molecular-mass biosurfactants are more effective at stabilizing oil-in-water emulsions [11,12]. Examples of biosurfactants and their producers are depicted in Table 1.

**Figure 1:**
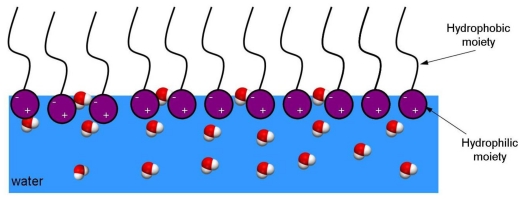
Accumulation of biosurfactants at the interface between liquid and air.

The biosurfactants accumulate at the interface between two immiscible fluids or between a fluid and a solid. By reducing surface (liquid-air) and interfacial (liquid-liquid) tension they reduce the repulsive forces between two dissimilar phases and allow these two phases to mix and interact more easily (Figure 1) [10].

**Figure 2:**
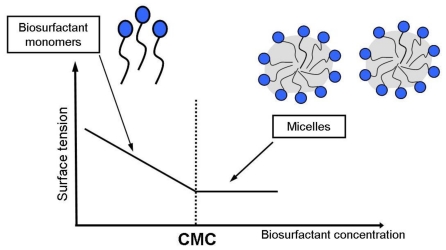
The relationship between biosurfactant concentration, surface tension and formation of micelles [14].

The most active biosurfactants can lower the surface tension of water from 72 to 30 mN·m^−1^ and the interfacial tension between water and *n*-hexadecane from 40 to 1 mN·m^−1^ [2,10]. Biosurfactant activities depend on the concentration of the surface-active compounds until the critical micelle concentration (CMC) is obtained. At concentrations above the CMC, biosurfactant molecules associate to form micelles, bilayers and vesicles (Figure 2). Micelle formation enables biosurfactants to reduce the surface and interfacial tension and increase the solubility and bioavailability of hydrophobic organic compounds [14]. The CMC is commonly used to measure the efficiency of surfactant. Efficient biosurfactants have a low CMC, which means that less biosurfactant is required to decrease the surface tension [2]. Micelle formation has a significant role in microemulsion formation [34]. Microemulsions are clear and stable liquid mixtures of water and oil domains separated by monolayer or aggregates of biosurfactants. Microemulsions are formed when one liquid phase is dispersed as droplets in another liquid phase, for example oil dispersed in water (direct microemulsion) or water dispersed in oil (reversed microemulsion) [2].

The biosurfactant effectiveness is determined by measuring its ability to change surface and interfacial tensions, stabilization of emulsions and by studying its hydrophilic-lipophilic balance (HLB). The HLB value is a measure to indicate whether a biosurfactant is related to water-in-oil or oil-in-water emulsion. This factor can be used to determine the suitable applicability of biosurfactants. Emulsifiers with low HLB are lipophilic and stabilize water-in-oil emulsification, whereas emulsifiers with high HLB have the opposite effect and confer better water solubility [2,35].

Biosurfactants also influence the bacterial cell surface hydrophobicity (CSH). This ability has been reported by Al-Tahhan *et al.* [36], who studied chemical and structural modifications in the CSH of *Pseudomonas aeruginosa* by a rhamnolipid in the presence of hexadecane. Results of their study demonstrated that rhamnolipid, at very low concentration, caused release of lipopolysaccharide (LPS) from the outer membrane resulting in an increase of cell surface hydrophobicity. In contrast, Sotirova *et al.* [37] reported that rhamnolipid at the concentrations below CMC did not affect the LPS component of the bacterial outer membrane but instead changed the composition of outer membrane proteins (OMP). However, all of the changes in the structure of the bacterial cell surface cause increase of accessibility of hydrocarbons to microbial cells.

## 3. Biosurfactants and Hydrocarbons Degradation/Remediation

The extensive production and use of hydrocarbons has resulted in widespread environmental contamination by these chemicals. Due to their toxicity, persistent and negative influence on living organisms it is important to clean-up the polluted sites. Hydrocarbons, as the hydrophobic organic chemicals, exhibit limited solubility in groundwater and tend to partition to the soil matrix. This partitioning can account for as much as 90–95% or more of the total contaminant mass. As a consequence, the hydrocarbon contaminants exhibit moderate to poor recovery by physico-chemical treatments; limited bioavailability to microorganisms; and limited availability to oxidative and reductive chemicals when applied to *in-situ* and/or *ex-situ* applications.

### 3.1. Role of Biosurfactants in Biodegradation Processes

**Figure 3:**
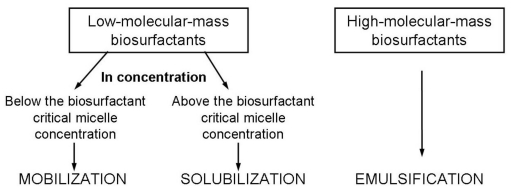
Mechanisms of hydrocarbon removal by biosurfactants depending on their molecular mass and concentration [11,42].

A promising method that can improve bioremediation effectiveness of hydrocarbon contaminated environments is the use of biosurfactants. They can enhance hydrocarbon bioremediation by two mechanisms. The first includes the increase of substrate bioavailability for microorganisms, while the other involves interaction with the cell surface which increases the hydrophobicity of the surface allowing hydrophobic substrates to associate more easily with bacterial cells [38]. By reducing surface and interfacial tensions, biosurfactants increase the surface areas of insoluble compounds leading to increased mobility and bioavailability of hydrocarbons. In consequence, biosurfactants enhance biodegradation and removal of hydrocarbons. Addition of biosurfactants can be expected to enhance hydrocarbon biodegradation by mobilization, solubilization or emulsification (Figure 3) [34,39–43].

The mobilization mechanism occurs at concentrations below the biosurfactant CMC. At such concentrations, biosurfactants reduce the surface and interfacial tension between air/water and soil/water systems. Due to the reduction of the interfacial force, contact of biosurfactants with soil/oil system increases the contact angle and reduces the capillary force holding oil and soil together. In turn, above the biosurfactant CMC the solubilization process takes place. At these concentrations biosurfactant molecules associate to form micelles, which dramatically increase the solubility of oil. The hydrophobic ends of biosurfactant molecules connect together inside the micelle while the hydrophilic ends are exposed to the aqueous phase on the exterior. Consequently, the interior of a micelle creates an environment compatible for hydrophobic organic molecules. The process of incorporation of these molecules into a micelle is known as solubilization [42].

Emulsification is a process that forms a liquid, known as an emulsion, containing very small droplets of fat or oil suspended in a fluid, usually water. The high molecular weight biosurfactants are efficient emulsifying agents. They are often applied as an additive to stimulate bioremediation and removal of oil substances from environments.

In the current literature, the latest advantages of the role of biosurfactants in interaction between hydrocarbons and microorganisms are presented. Franzetti *et al.* [17] describe proposed roles for biosurfactants with respect to their interactions between microorganisms and hydrocarbons in the content of modulation of cell surface hydrophobicity. High cell-hydrophobicity allows microorganisms to directly contact oil drops and solid hydrocarbons while low cell hydrophobicity permits their adhesion to micelles or emulsified oils [17]. They discuss three mechanisms of interaction between microorganisms and hydrocarbons: access to water-solubilized hydrocarbons, direct contact of cells with large oil drops and contact with pseudosolubilized or emulsified oil. The authors suggest that during the different growth stages of microorganisms, biosurfactants can change hydrocarbon accession modes. In their studies, they observed that *Gordonia* sp. strain BS 29 grown on hydrocarbons produced cell-bound glycolipid biosurfactant and extracellular bioemulsifier, and during the phase of the growth on hexadecane the surface hydrophobicity changes were observed [17,44].

The recent report by Cameotra and Singh [45] throws more light on the uptake mechanism of *n*-alkane by *Pseudomonas aeruginosa* and the role of rhamnolipids in the process. The authors reported a new and exciting research for hydrocarbon uptake involving internalization of hydrocarbon inside the cell for subsequent degradation. Biosurfactant action dispersed hexadecane into microdroplets, increasing the availability of the hydrocarbon to the bacterial cells. The electron microscopic studies indicated that uptake of the biosurfactant-coated hydrocarbon droplets occurred. Interestingly, “internalization” of “biosurfactant layered hydrocarbon droplets” was taking place by a mechanism similar in appearance to active pinocytosis. This mechanism was not earlier visually reported in bacterial modes for hydrocarbon uptake. Although much work has been done by many groups to explain the role of biosurfactants in the degradation of water immiscible substrates, most processes still remain unclear.

### 3.2. Biodegradation Studies

The capability of biosurfactants and biosurfactant-producing bacterial strains to enhance organic contaminants’ availability and biodegradation rates was reported by many authors [39,41,46]. Obayori *et al.* [47] investigated the biodegradative properties of biosurfactant produced by *Pseudomonas* sp. LP1 strain on crude oil and diesel. The results obtained confirmed the ability of strain LP1 to metabolize the hydrocarbon components of crude and diesel oil. They reported 92.34% degradation of crude oil and 95.29% removal of diesel oil. Biodegradative properties of biosurfactant producing *Brevibacterium* sp. PDM-3 strain were tested by Reddy *et al.* [48]. They reported that this strain could degrade 93.92% of the phenanthrene and also had ability to degrade other polyaromatic hydrocarbons such as anthracene and fluorene.

Kang *et al.* [49] used sophorolipid in studies on biodegradation of aliphatic and aromatic hydrocarbons and Iranian light, crude oil under laboratory conditions. Addition of this biosurfactant to soil increased also biodegradation of tested hydrocarbons with the rate of degradation ranging from 85% to 97% of the total amount of hydrocarbons. Their results indicated that sophorolipid may have potential for facilitating the bioremediation of sites contaminated with hydrocarbons having limited water solubility and increasing the bioavailability of microbial consortia for biodegradation.

The effective microbiological method in bioremediation of hydrocarbon polluted sites is the use of biosurfactant producing bacteria without necessarily characterizing the chemical structure of the surface active compounds. The cell free culture broth containing the biosurfactants can be applied directly or by diluting it appropriately to the contaminated site. The other benefit of this approach is that the biosurfactants are very stable and effective in the culture medium that was used for their synthesis.

The usefulness of biosurfactant producing strains in bioremediation of sites highly contaminated with crude petroleum-oil hydrocarbons was confirmed by Das and Mukherjee [50]. The ability of three biosurfactant producing strains: *Bacillus subtilis* DM-04, *Pseudomonas aeruginosa* M and *Pseudomonas aeruginosa* NM to remediate petroleum crude-oil contaminated soil samples was investigated by treating the soil samples with aqueous solutions of biosurfactants obtained from the respective bacteria strains. Additionally, the tested soil was inoculated with mineral-salts media containing a specified amount of *Bacillus subtilis* DM-04 or *Pseudomonas aeruginosa* M and NM strains. To determine the extent of biodegradation, the soil-phase total petroleum hydrocarbons (TPH) concentrations were analyzed after 120 days and compared to a control where the soil was treated with un-inoculated medium. Bioagumentation of studied soil with *P. aeruginosa* M and NM consortium and *B. subtilis* strain showed that TPH levels were reduced from 84 to 21 and 39 g·kg^−1^ of soil, respectively. In contrast, the TPH level was decreased to 83 g·kg^−1^ in control soil.

Joseph and Joseph [51] separated the oil from the petroleum sludge by induced biosurfactant production by bacteria. Petroleum sludge is generated in significant amount in the refineries during crude oil processing. Crude oil is usually stored in storage tanks. Pollutants present in the oil are deposited at bottom of the tank. During cleaning of the tank the sludge is recovered and is treated as a waste. The sludge used for the investigation contained TPH in the concentration range of 850 ± 150 g·kg^−1^. In this study the sludge was inoculated directly with *Bacillus* sp. strains and by addition of the cell free supernatant. Un-inoculated sludge was also taken as a control. Upon inoculation of the supernatant to the sludge slurry, oil separation and reduction of TPH was observed. The oil separation process was slow initially in the test supplied with the fresh inoculation of the bacterium compared to the samples inoculated with the supernatant, but the residual TPH of both became equal within 48 h. The efficiency of removal of the various isolates ranged from 91.67% to 97.46%. Therefore, it has been observed that the biosurfactant produced by the primary inoculum remained in the supernatant and it was enough to continue the reaction. The biosurfactant displayed the property to reduce surface and interfacial tensions in both aqueous and hydrocarbon mixtures and hence had potential for oil recovery.

Biosurfactants have often been used to enhance bioavailability and biodegradation of hydrophobic compounds but there is little knowledge available about the effect of simultaneous emulsifier production on biodegradation of complex hydrocarbon mixtures. Nievas *et al.* [43] studied the biodegradation of a bilge waste which is a fuel oil-type complex residue produced in normal ship operations. Bilge waste is a hazardous waste composed of a mixture of sea-water and hydrocarbon residue, where *n*-alkanes, resolvent total hydrocarbons and unsolvent complex mixture are the main constituents. Unsolvent complex mixture principally is composed by branched and cyclic aliphatic hydrocarbons and aromatic hydrocarbons, which usually show the greatest resistance to biodegradation. In their studies, they investigated the biodegradation of an oily bilge wastes by an emulsifier-producing microbial consortium. As the result for both levels of oily wastes, 136 g·kg^−1^ of resolvent hydrocarbons and 406 g·kg^−1^ of unsolvent mixture, they found that all of the hydrocarbon types showed an important concentration reduction from their initial values. They observed that the extent of biodegradation followed the order *n*-alkanes > resolved total hydrocarbon > unsolvent complex mixture. An emulsifier-producing microbial consortium used for biodegradation of bilge wastes showed reduction of *n*-alkanes, resolvent hydrocarbons and unsolvent mixture around by 85%, 75% and 58%, respectively.

Barkay *et al.* [52] tested the effect of a bioemulsifier alasan produced by *Acinetobacter radioresistens* KA53 on the solubilization of polyaromatic hydrocarbons (PAHs), phenanthrene (PHE) and fluoranthene (FLA). They also studied the influence of alasan on mineralization of PHE and FLA by *Sphingomonas paucimobilis* EPA505. They indicated that aqueous solubility of phenanthrene and fluoranthene increased linearly in the presence of increasing concentrations of bioemulsifier (50 to 500 μg·mL^−1^) and mineralization of PAHs by *S. paucimobilis* EPA505 was stimulated by appearance of alasan. The presence of alasan at concentrations of up to 300 μg·mL^−1^ more than doubled the degradation rate of fluoranthene and significantly increased the degradation rate of phenanthrene. Increasing the alasan concentration over 300 μg·mL^−1^ had no further stimulation on PAHs mineralization, although solubilization curves showed that the apparent solubility of these compounds continued to increase linearly with alasan additions in this concentration range. This could be explained by association of PAHs with multimolecular structures of alasan, formed at concentrations above the CMC (about 200 μg·mL^−1^), which was not readily available for the degrading strain.

Martínez-Checa *et al.* [53] investigated the usefulness of the V2-7 bioemulsifier producing strain F2-7 of *Halomonas eurihalina* in oil bioremediation process. First, they studied capacity of strain F2-7 to grow and produce bioemulsifier in the presence of different hydrocarbon compounds. They observed that all analyzed hydrocarbons supported the growth of F2-7 strain and the production of V2-7 bioemulsifier. The ability of the analyzed strain to remove polycyclic aromatic hydrocarbons was investigated during the growth of this strain for 96 h in liquid medium supplemented with naphthalene, phenanthrene, fluoranthene and pyrene. After the experiment, the obtained residual concentrations of fluoranthene (56.6%) and pyrene (44.5%) were higher than naphthalene (13.6%) and phenanthrene (15.6%). Efficiency of strain F2-7 in removing PAHs confirmed its potential applicability in oil bioremediation technology.

Exopolysaccharide (EPS) secreted by *Enterobacter cloacae* strain TU was also reported as an emulsifier [54]. EPS was investigated and was found to have a high emulsifying activity (*E*_24_ = 75). The EPS could increase the hydrophobicity of the bacterial cell surface and also neutralize the surface charge of the cells.

### 3.3. Soil Washing Technology

Soil washing technology is characterized by chemico-physical properties of the biosurfactant and not by their effect on metabolic activities or changes in cell-surface properties of bacteria [55]. However, the processes may enhance the bioavailability for bioremediation. Aqueous solutions of biosurfactants can be also used to release compounds characterized by low solubility from soil and other media in process called washing.

Urum *et al.* [56] investigated the efficiency of different surfactant solutions in removing crude oil from contaminated soil using a soil washing process. They demonstrated higher crude oil elimination by synthetic surfactant-sodium dodecyl sulfate (SDS) and rhamnolipid biosurfactants (46% and 44%, respectively) than natural surfactants—saponins (27%).

Kang *et al.* [49] analyzed application of sophorolipid, Tween 80/60/20 and Span 20/80/85 as possible soil washing agents to release 2-methylnapthalene from artificially polluted soil. They observed that sophorolipid had a higher soil washing efficiency that any other tested nonionic surfactants except Tween 80. This could be caused by high hydrophilic-lipophilic balance (HLB) of Tween 80. It appeared that surfactants with a higher HLB resulted in better solubility of 2-methylnapthalene.

Lai *et al.* [57] studied the ability of removing total petroleum hydrocarbon (TPH) from soil by two biosurfactants: rhamnolipid and surfactin, and two synthetic surfactants: Tween 80 and Triton X-100. The TPH removal efficiency was examined for low TPH-contaminated (LTC) and high TPH-contaminated (HTC) soils (containing 3000 and 9000 mg·kg^−1^ dry soil of TPH, respectively) by washing them with (bio) surfactant solutions. As a result, they observed that addition of 0.2 mass% of rhamnolipid, surfactin, Triton X-100 and Tween 80 to LTC soil resulted in a TPH removal of 23%, 14%, 6% and 4%, respectively, while for HTC soil a significantly higher TPH removal efficiency of 63%, 62%, 40% and 35%, respectively, was observed. These results indicated that among four (bio) surfactants, rhamnolipid and surfactin showed superior performance on TPH removal, compared to synthetic surfactants. The two biosurfactants examined in this work have the potential to be used as biostimulation agents for bioremediation of oil-polluted soils.

Franzetti *et al.* [44] evaluated the application of surface active compounds produced by *Gordonia* sp. strain BS29 in soil remediation technologies: bioremediation of soils contaminated by aliphatic and aromatic hydrocarbons (microcosm bioremediation experiment), and washing of soils contaminated by crude oil, PAHs, and heavy metals (batch experiment). The work represents the first study on the potential applications of surface-active compounds produced by *Gordonia* sp. in environmental remediation techniques for contaminated soils. In the previous work, surface-active compounds produced by *Gordonia* sp. and their role in the access to hydrocarbons were characterized [58]. The bacterial strain grew on aliphatic hydrocarbons and produced two different types of surface active compounds: extracellular bioemulsan and cell-bound biosurfactant. Bioremediation results showed that the bioemulsans produced by *Gordonia* sp. strain BS29 were able to slightly enhance the biodegradation of recalcitrant branched hydrocarbons. On the other hand, the authors obtained the best results in soil washing of hydrocarbons. The mean of the crude oil removal for bioemulsans was 33%. The study presented by Franzetti *et al.* [58] showed that the BS29 bioemulsans from *Gordonia* sp. are promising washing agents for remediation of hydrocarbon-contaminated soils. The BS29 bioemulsans were also able to remove metals (Cu, Cd, Pb, Zn, Ni), but their potential in the process was lower than rhamnolipids.

### 3.4. Clean-up Combined Technology

The aim of the research work reported by Kildisas *et al.* [59] and Baskys *et al.* [60] was to develop inexpensive and efficient combined (complex) technology for cleaning up the soil contaminated by oil pollutants in a large scale. The described technology was based on bioremediation or phytoremediation principles and used physical-chemical treatment by washing the contaminated soil. The complex technology consisted of two stages: at the first stage, the migrating fraction of pollutants was separated from soil using biosurfactants; at the second stage, the remaining not migrating fraction was rendered harmless using biodegradation. Phytoremediation was also applied to enhance soil quality. The completed clean up complex technology is presented by Kildisas *et al.* [59]. The presented technology consisted of washing of the migration fraction by application of biosurfactants, separation of water, oil and soil, biodegradation of residual non-migrating oil fraction by use of specific bacteria with potential to degrade the crude oil and oil products, and phytoremediation. The pilot plant for washing the contaminated soil was designed and constructed in a space of 340 m^2^ in which 1000 m^3^ of contaminated soil was cleaned up. In the beginning of the pilot experiment the concentrations of the oil pollutants were between 180– 270 g·kg^−1^ of soil, and after washing the concentrations were reduced to 34–59 g·kg^−1^ of soil. After degradation, the pollutant concentrations dropped to 3.2–7.3 g·kg^−1^ of soil [60].

### 3.5. Microbial Enhanced Oil Recovery (MEOR)

#### 3.5.1. Mechanism of MEOR

**Figure 4:**
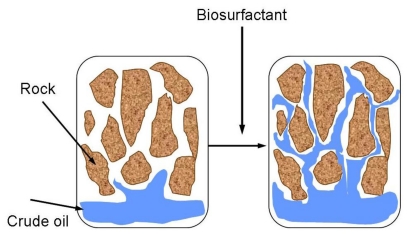
Mechanism of enhanced oil recovery by biosurfactants.

Biosurfactants can also be involved in microbial enhanced oil recovery (MEOR). MEOR methods are used to recover oil remaining in reservoirs after primary (mechanical) and secondary (physical) recovery procedures [61,62]. It is an important tertiary process where microorganisms or their metabolites, including biosurfactants, biopolymers, biomass, acids, solvents, gases and also enzymes, are used to increase recovery of oil from depleted reservoirs. Application of biosurfactants in enhanced oil recovery is one of the most promising advanced methods to recover a significant proportion of the residual oil. The remaining oil is often located in regions of the reservoir that are difficult to access and the oil is trapped in the pores by capillary pressure [62]. Biosurfactants reduce interfacial tension between oil/water and oil/rock. This reduces the capillary forces preventing oil from moving through rock pores (Figure 4). Biosurfactants can also bind tightly to the oil-water interface and form emulsion. This stabilizes ^−^the desorbed oil in water and allows removal of oil along with the injection water [63].

#### 3.5.2. Applications of MEOR

Bordoloi and Konwar [64] investigated the recovery of crude oil from a saturated column under laboratory conditions. Laboratory studies on MEOR typically utilize core substrates and columns containing the desired substrate, usually sand. This substrate is used to demonstrate the usefulness of biosurfactants in recovery of oil from reservoirs. For this purpose, a glass column is packed with dry sand, then the column is saturated with crude oil and aqueous solution of biosurfactant is poured in the column. The potential of biosurfactants in MEOR is estimated by measuring the amount of oil released from the column after pouring the aqueous solution of biosurfactant in the column. The experiment was carried out in room temperature, 70 and 90 °C to evaluate the influence of temperature on biosurfactant-induced oil recovery. Biosurfactants used in the experiment were produced by bacterial isolates of *P. aeruginosa* strains (MTCC7815, MTCC7814, MTCC7812 and MTCC8165). Biosurfactants of MTCC7815, MTCC7812 and MTCC8165 strains recovered about 49–54% of crude oil from the sand packed column at room temperature; 52–57% at 70 °C and 58–62% at 90 °C. The biosurfactant produced by MTCC7814 was reported to be less efficient. In control samples treated with culture medium, very little recovery of crude oil was obtained.

Jinfeng *et al.* [65] evaluated the technical feasibility and effectiveness of improving oil recovery by microbial enhanced water-flooding techniques in high temperature petroleum reservoirs. The studies were conducted in Guan 69 Unit in Dagang Oilfield in China by injection of a mixture of *Arthrobacter* sp. (A02), *Pseudomonas* sp. (P15) and *Bacillus* sp. (B24) strain suspension and the nutrient solution through injection wells in an ongoing waterflood reservoir where the temperature reached 73 °C. The pattern of injection “nutrient-suspension-nutrient” was designed based on the knowledge of the reservoir conditions and the mechanism of enhancement of oil recovery by the selected strains in the reservoir. The oil production performance in the unit was periodically monitored before, during and after microbial water-flooding and then compared. Jinfeng *et al.* [65] observed that the oil production steadily increased after microbial water-flooding. The oil production in the unit before and in the beginning phase of the injection decreased from 55 t/day in January 2000 to 30 t/day in September 2001, which implies a decline rate of 21%. This situation changed markedly six month later and by the end of the July 2004, about 8700 t of additional oil was obtained compared with the predicted oil production. All the seven production wells showed a positive response to the treatment, of which five wells evidently increased in oil production.

Pornsunthorntawee *et al.* [66] compared the oil recovery activities of the biosurfactants produced by *Bacillus subtilis* PT2 and *Pseudomonas aeruginosa* SP4 with three synthetic surfactants: polyoxyethylene sorbitan monooleate (Tween 80), sodium dodecyl benzene sulfonate (SDBS) and sodium alkyl polypropylene oxidebsulfate (Alfoterra). For this purpose, sand-packed column inoculated with a motor oil complex was used. The surfactant solutions were poured onto the packed column to test their ability to enhanced oil recovery. The results showed that the biosurfactants produced by *Bacillus subtilis* PT2 and *Pseudomonas aeruginosa* SP4 were more efficient in oil recovery, removing about 62% and 57%, respectively, of the tested oil. The biosurfactants produced by *Bacillus subtilis* PT2 could recover oil more effectively than that produced by *Pseudomonas aeruginosa* SP4. In the case of tested synthetic surfactants, the oil recovery was found to be approximately 53–55%.

Biosurfactants can also be used to extract hydrocarbon compounds from oil shales in order to utilize it as a substitute for petroleum energy fuel. In studies conducted by Haddadin *et al.* [67], biosurfactants produced by *Rhodococcus erythropolis* and *Rhodococcus ruber* were successfully used for desorption of the hydrocarbons from El-Lajjun oil shale.

## 4. Biosurfactants and Metals Remediation

Contamination of soil environments with heavy metals is very hazardous for human and other living organisms in the ecosystem. Due to their extremely toxic nature, presence of even low concentrations of heavy metals in the soils has been found to have serious consequences. Nowadays, there are many techniques used to clean up soils contaminated with heavy metals. Remediation of these soils includes non biological methods such as excavation, and disposal of contaminated soil to landfill sites or biological techniques [68]. Biological methods are processes that use plants (phytoremedation) or microorganisms (bioremediation) to remove metals from soil. Application of microorganisms was discovered many years ago to help in reduction of metal contamination. Heavy metals are not biodegradable; they can only be transferred from one chemical state to another, which changes their mobility and toxicity. Microorganisms can influence metals in several ways. Some forms of metals can be transformed either by redox processes or by alkylation. Metals can also be accumulated by microorganisms by metabolism-independent (passive) or by intracellular, metabolism-dependent (active) uptake. Microorganisms can influence metal mobility indirectly by affecting pH or by producing or releasing substances which change mobility of the metals [69,70].

Two following methods, “soil washing” or “soil flushing”, are involved in remediation of metal contaminated soil. The first technique used is *ex situ*—contaminated soil is excavated, put into the glass column and washed with biosurfactant solution. In turn, soil flushing of *in situ* technologies involves use of drain pipes and trenches for introducing and collecting biosurfactant solution to and from the soil [15,71]. Interestingly, biosurfactants can be used for metal removal from the soil. Biosurfactants can be applied to a small part of contaminated soil in which soil is put in a huge cement mixer, biosurfactant-metal complex is flushed out, soil deposited back, and biosurfactant-metal complex treated to precipitate out biosurfactant, leaving behind the metal. The bond formed between the positively charged metal and the negatively charged surfactant is so strong that flushing water through soil removes the surfactant metal complex from the soil matrix. This method can also be carried out for deeper subsurface contamination only with more pumping activities.

### 4.1. Removal of Metals by Biosurfactants—Mechanism of the Process

Using biosurfactants have unquestionable advantages because bacterial strains able to produce surface active compounds do not need to have survival ability in heavy metal-contaminated soil. However, using biosurfactants alone requires continuous addition of new portions of these compounds.

**Figure 5:**
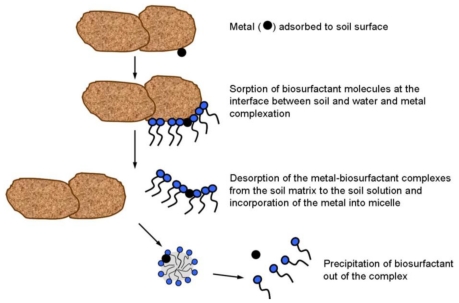
Mechanism of biosurfactant activity in metal-contaminated soil [74].

The usefulness of biosurfactants for bioremediation of heavy metal contaminated soil is mainly based on their ability to form complexes with metals. The anionic biosurfactants create complexes with metals in a nonionic form by ionic bonds. These bonds are stronger than the metal’s bonds with the soil and metal-biosurfactant complexes are desorbed from the soil matrix to the soil solution due to the lowering of the interfacial tension. The cationic biosurfactants can replace the same charged metal ions by competition for some but not all negatively charged surfaces (ion exchange). Metal ions can be removed from soil surfaces also by the biosurfactant micelles. The polar head groups of micelles can bind metals which mobilize the metals in water (Figure 5) [38,71–73].

#### 4.1.2. Applications of the Process

Biosurfactants which are used in bioremediation of metal-contaminated soils have been proposed for use in metal removal in recent years [72,73]. High potential of biosurfactants in mobilization and decontamination of heavy metal contaminated soil was confirmed by Juwarkar *et al.* [75], who used di-rhamnolipid biosurfactant produced by *Pseudomonas aeruginosa* BS2 for mobilization of metals from multi-metal contaminated soil. To study the feasibility of di-rhamnolipid to remove chromium, lead, cadmium and copper from soil, a column study was conducted. Heavy metal spiked soil into a glass column was washed with 0.1% di-rhamnolipid biosurfactant solution. The results indicated that di-rhamnolipid selectively removed heavy metals from soil in the order of Cd = Cr > Pb = Cu > Ni.

In turn, Das *et al.* [76] investigated the possibility of using the biosurfactant produced by marine bacterium for removal of heavy metals from solutions. The positive role of marine biosurfactant in the remediation of polyaromatic hydrocarbons was reported earlier [7], however there was no information about the role of this biosurfactant in heavy metal remediation. The study revealed that tested anionic biosurfactant was able to bind the metal ions and the percentage removal of Pb and Cd metals varied with the different concentrations of metals and biosurfactants. The ability of biosurfactant of marine origin to chelate toxic heavy metals and form an insoluble precipitate could be useful in treatment of heavy metal containing wastewater.

Removal of heavy metals from sediments could be enhanced by use of solution containing biosurfactant and inorganic compounds. For example, Dahrazma and Mulligan [77] reported the higher rate of removal of copper and nickel from sediments by adding 1% NaOH to the solution of rhamnolipid. Many metals mostly exist in the environment organic fraction, adding OH^−^ to the sediment solubilizes this fraction, and thus, more metals are available for removal by a rhamnolipid biosurfactant.

Another effective method for the remediation of heavy metals contaminated soil is biosurfactant foam technology. Wang and Mulligan [78] evaluated the feasibility of using rhamnolipid foam to remove Cd and Ni from a sandy soil. They reported that the use of foam had a significant effect on the mobility of biosurfactant flowing in a porous medium and made a more uniform and efficient contact of biosurfactant with the metals. Application of rhamnolipid foam increases efficiency and allows removal of 73.2% and 68.1% of Cd and Ni, respectively, whereas the rhamnolipid solution flushed only 61.7% and 51% of Cd and Ni, respectively. The system used for the experiment is presented schematically by Wang and Mulligan [78].

The rate of heavy metal removal from soil strongly depends on its chemical composition. The predominant constituent of the sand and silt fraction in many soils is quartz, thus quartz was chosen for the bioremediation experiment. Aşçi *et al.* [68] studied recovery of the metal ions from quartz by rhamnolipid. They observed that the best recovery efficiency from quartz, approximately 91.6% of the sorbed Cd and 87.2% of the sorbed Zn, was achieved using 25 mM rhamnolipid concentration.

Biosurfactants were also used to evaluate their potential in arsenic mobilization from the mine tailings [79]. The experimental results showed that introduction of rhamnolipid enhanced As mobilization from the mine tailings significantly. The mobilization increased with the concentration of biosurfactant and became relatively stable when the concentration of rhamnolipid was above 100 mg·L^−1^. It has been reported by Doong *et al.* [80] that the removal of heavy metals increased linearly with increasing surfactant concentration below the CMC and remained relatively constant above the CMC. The CMC of the biosurfactant used by Wang and Mulligan [79] was around 30 mg·L^−1^. The high concentration of rhamnolipid required in this experiment could be due to the sorption of biosurfactant to the mine tailings and the dilution and binding effects of mine tailing particles. The biosurfactant may be enhancing As mobilization by reducing the interfacial tension between As and the mine tailings, by formation of aqueous complexes or micelles and by improving the wettability of the mine tailings. The results from this research study indicated that biosurfactants have potential to be used in the remediation of As-contaminated mine tailings and they can be also effectively used to remove As from soils.

Besides the mobilization, biosurfactants can be involved in other processes connected with remediation of heavy metals. They are used, for example, in entrapping of trivalent chromium in micelles which provides bacterial tolerance and resistance towards high concentration of Cr(III). Gnanamani *et al.* [81] studied the bioremediation of chromium (VI) by biosurfactant producing, marine isolate *Bacillus* sp. MTCC 5514. The remediation carried out by this strain proceeded via two processes: reduction of Cr(VI) to Cr(III) by extracellular chromium reductase and entrapment of Cr(III) by the biosurfactants. The first process transforms the toxic state of chromium into less-toxic state and the second process prevents the bacterial cells from the exposure of chromium(III). Both reactions keep bacterial cells active all the time and provide tolerance and resistance toward high hexavalent and trivalent chromium concentrations.

### 4.2. Biosurfactants and Phytoremediation

Efficiency of phytoremediation of heavy metal contaminated soils can be increased by inoculation of plants by biosurfactant-producing and heavy metal-resistant bacteria. Biosurfactant-producing *Bacillus* sp. J119 strain was investigated for its capability to promote the plant growth and cadmium uptake of rape, maize, sudangrass and tomato in soil contaminated with different levels of Cd [82]. The study demonstrated that the tested strain could colonize the rhizosphere of all studied plants but its application enhanced biomass and Cd uptake only in plant tissue of tomato. This means that root colonization activity of the introduced strain is plant type influenced. However, further analyses of interactions between the plants and biosurfactant-producing bacterial strain J119 may provide a new microbe assisted-phytoremediation strategy for metal-polluted soils. Further work on the applications of biosurfactants and biosurfactants-producing bacteria in phytoremediation, especially in sites co-contaminated with organic and metal pollutants is required.

## 5. Biosurfactants in Co-Contaminated Sites Remediation

It was estimated by the U.S. Environmental Protection Agency that 40% of sites are co-contaminated with organics and metals pollutions [83]. The presence of toxic metals (lead, cadmium, arsenic) in some cases causes inhibition of organic compound biodegradation [83–85]. However, a review of the literature shows a number of possible approaches that can lower metal bioavailability and/or increase microbial tolerance to metals. These include inoculation with metal-resistant microorganisms, addition of materials like: clay minerals—kaolinite and montmorillonite, calcium carbonate, phosphate, chelating agents (EDTA), and bio- and surfactants [83]. Biosurfactants produced by microorganisms show promise for enhancing organic compound biodegradation in the presence of metals. Application of biosurfactants or microorganism produced biosurfactants in *in situ* co-contaminated sites bioremediation seems to be more environmentally compatible and more economical than using modified clay complexes or metal chelators.

Sandrin *et al.* [84] showed that metal-complexing rhamnolipids reduced metal toxicity to allow enhanced organic biodegradation by *Burkholderia* sp. under laboratory conditions. This research demonstrated that rhamnolipids induced the release of lipopolisaccharide (LPS) from gram-negative bacteria, *Burkholderia* sp., which does not produce rhamnolipid. The authors suggested that rhamnolipid was able to reduce metal toxicity to microbial consortia in co-contaminated soils through a combination of metal complexation and in the alteration of cell surface properties through the release of lipopolisaccharide (LPS), resulting in enhanced bioremediation effect. Maslin and Maier [85] studied the effect of rhamnolipids produced by various *Pseudomonas aeruginosa* strains on the phenanthrene degradation by indigenous populations in two soils co-contaminated with phenanthrene and cadmium. The authors showed that rhamnolipids applied had the ability to complex cationic metals, increasing the phenanthrene bioavailability [85]. The biodegradation of phenanthrene was increased from 7.5 to 35% in one soil, and from 10 to 58% in the second soil, in response to rhamnolipids application. As biosurfactants are degraded by soil populations in 2–3 weeks, Maslin and Maier [85] used a pulsing strategy, in which new portions of rhamnolipids were added to the system to maintain a constant level of biosurfactant during organic contaminant mineralization.

## 6. Conclusions and Future Perspectives

Application of biosurfactant and biosurfactant-producing bacteria in environmental technologies (bioremediation and phytoremediation) has been studied. Both organic and inorganic contaminants can be removed through different processes (physico-chemical and biological) in which biosurfactants are involved. Due to their biodegradability and low toxicity, they are very promising for use in environmental biotechnologies. The commercial success of biosurfactants is still limited by their high production cost. Optimized growth conditions using cheap renewable substrates (agro-industrial wastes) and novel, efficient methods for isolation and purification of biosurfactants could make their production more economically feasible. Another important aspect regarding biological remediation technologies is the use of biosurfactant in the process on a large scale. To felicitate this process, new techniques should be developed such as foams or micro-foams (colloidal gas aphrons-CGA) in conjunction with biosurfactants.

Little is known about the potential of biosurfactant production by microorganisms *in situ*. Most of the described studies were done under laboratory conditions. More efforts are required to evaluate the biosurfactant production by microorganisms *in situ* and their role in biological remediation technologies. Remediation systems with only one type of the contaminant have been studied to gain a basic understanding. Only a few studies have also been completed on metal-organic pollutant co-contaminated site remediation [86]. More information is required concerning the structures of biosurfactants, their interaction with soil and contaminants and scale up and cost effective biosurfactant production [86]. For lowering the cost of biosurfactant production, commercially viable biological and engineering solutions are required. One important point in this context is the use of low cost substrates for production of biosurfactants.

A promising approach seems to be the application of inoculants of biosurfactant producing bacteria in phytoremediation of hydrocarbon polluted soil to improve the efficiency of this technology. Application of the biosurfactants in phytoremediation on a large scale requires studies to identify their potential toxic effect on plants. Although the biosurfactants are thought to be ecofriendly, some experiments indicated that under certain circumstances they can be toxic to the environment [87]. Nevertheless, careful and controlled use of these interesting surface active molecules will surely help in the enhanced clean up of the toxic environmental pollutants and provide us with a clean environment.

## Figures and Tables

**Table 1:** Classification of biosurfactants and their use in remediation of heavy metal and hydrocarbon contaminated sites.

Biosurfactant		Microorganism	Applications in Environmental Biotechnology	References
Group	Class			
**Glycolipids**	Rhamnolipids	*Pseudomonas aeruginosa*, *Pseudomonas* sp.	Enhancement of the degradation and dispersion of different classes of hydrocarbons; emulsification of hydrocarbons and vegetable oils; removal of metals from soil	[13–16]
	Trehalolipids	*Mycobacterium tuberculosis*, *Rhodococcus erythropolis*, *Arthrobacter* sp., *Nocardia* sp., *Corynebacterium* sp.	Enhancement of the bioavailability of hydrocarbons	[17]
	Sophorolipids	*Torulopsis bombicola*, *Torulopsis petrophilum*, *Torulopsis apicola*	Recovery of hydrocarbons from dregs and muds; removal of heavy metals from sediments; enhancement of oil recovery	[14,18,19]
**Fatty acids, phospholipids and neutral lipids**	Corynomycolic acid	*Corynebacterium lepus*	Enhancement of bitumen recovery	[20]
	Spiculisporic acid	*Penicillium spiculisporum*	Removal of metal ions from aqueous solution; dispersion action for hydrophilic pigments; preparation of new emulsion-type organogels, superfine microcapsules (vesicles or liposomes), heavy metal sequestrants	[21–23]
	Phosphati-dylethanolamine	*Acinetobacter* sp., *Rhodococcus erythropolis*	Increasing the tolerance of bacteria to heavy metals	[24]
**Lipopeptides**	Surfactin	*Bacillus subtilis*	Enhancement of the biodegradation of hydrocarbons and chlorinated pesticides; removal of heavy metals from a contaminated soil, sediment and water; increasing the effectiveness of phytoextraction	[25–27]
	Lichenysin	*Bacillus licheniformis*	enhancement of oil recovery	[28]
**Polymeric biosurfactants**	Emulsan	*Acinetobacter calcoaceticus* RAG-1	Stabilization of the hydrocarbon-inwater emulsions	[29]
	Alasan	*Acinetobacter radioresistens* KA-53		[30]
	Biodispersan	*Acinetobacter calcoaceticus* A2	Dispersion of limestone in water	[31]
	Liposan	*Candida lipolytica*	Stabilization of hydrocarbon-in-water emulsions	[32]
	Mannoprotein	*Saccharomyces cerevisiae*		[33]
